# Turning sex inside-out: Peripheral contributions to sexual differentiation of the central nervous system

**DOI:** 10.1186/2042-6410-3-12

**Published:** 2012-05-28

**Authors:** Ashlyn Swift-Gallant, Lee Niel, D Ashley Monks

**Affiliations:** 1 Department of Psychology, University of Toronto Mississauga, Mississauga, ON, Canada; 2 Department of Population Medicine, University of Guelph, Guelph, ON, Canada; 3 Cell and Systems Biology, University of Toronto, Toronto, ON, Canada; 4 Institute of Medical Sciences, University of Toronto, Toronto, ON, Canada; 5 Neuroscience Program, University of Toronto, AA, ON, Canada

**Keywords:** Brain, Spinal cord, Sexual differentiation, Androgen receptor, Spinal nucleus of the bulbocavernosus, Neuromuscular, Sexual behavior

## Abstract

Sexual differentiation of the nervous system occurs via the interplay of genetics, endocrinology and social experience through development. Much of the research into mechanisms of sexual differentiation has been driven by an implicit theoretical framework in which these causal factors act primarily and directly on sexually dimorphic neural populations within the central nervous system. This review will examine an alternative explanation by describing what is known about the role of peripheral structures and mechanisms (both neural and non-neural) in producing sex differences in the central nervous system. The focus of the review will be on experimental evidence obtained from studies of androgenic masculinization of the spinal nucleus of the bulbocavernosus, but other systems will also be considered.

## Review

One of the most vexing problems facing those who study sexual differentiation of the nervous system was apparent right at the beginning. Phoenix and colleagues [1] are widely credited with founding this field of study by demonstrating that sex hormones, when manipulated prenatally and in adulthood, can reverse sex-typical copulatory behaviors in guinea pigs. Notably, although the authors identified the nervous system as the *likely* site of sex hormone action, they acknowledged that in principle the observed effects on behavior could result from actions on non-neural tissues. We now have overwhelming evidence that early gonadal hormones do indeed organize sex differences in the central nervous system (CNS), and we believe that these sexual dimorphisms contribute to sex differences in behavior [2-6]. Nonetheless, we are still forced to make a similarly qualified statement about our current state of knowledge of site of androgen action in organizing behavior, precisely *because* there is good evidence for the periphery, including the peripheral nervous system as well as non-neural tissues (e.g. muscle), contributing to sex differentiation of the CNS [7]. The goal of providing a satisfying description of the mechanisms whereby sex hormones produce *any* of the known sex differences in the nervous system has remained elusive despite intensive investigation. Nonetheless, to the extent to which we have answers to the site of action question, there appear to be as many or more cases of indirect actions of steroid hormones on the CNS via peripheral structures.

### The spinal nucleus of the bulbocavernosus (SNB)

The SNB is a neuromuscular system that mediates copulatory functions associated with the phallus. When the the SNB system is disabled via target muscle ablation, intense reflexive erection of the glans penis known as “cups”, efficient ejaculation, and the efficient deposition and removal of seminal plugs are impaired [8-11]. These erectile and ejaculatory functions of the SNB system are critical for male fertility, perhaps explaining the strong conservation of the SNB system among mammals [6].

In rats, both SNB motoneurons and their target muscles are larger in males than females due to testosterone action both during a perinatal sensitive period [12-14] as well as throughout adult life [15,16]. Unlike many CNS dimorphisms, estrogenic metabolites of testosterone play only a minor role in its organization [17,18], whereas activation of androgen receptors (ARs) is both necessary [15,19] and largely sufficient [12,20] for masculinization of the SNB. Because AR is expressed in many cells, including SNB motoneurons [21-24] and target muscles [25,26], it is unclear whether testosterone acts on motoneurons, muscles or other cells to masculinize the system. The SNB is also unusual in that, as a neuromuscular system, the targets of SNB neurons are known and are relatively accessible for study, and experiments in which the components of this system are dissociated are feasible. As a result, the rodent SNB is the CNS sexual dimorphism in which site of action has been addressed most extensively. It is worth noting that when some of these conditions have been met in other CNS dimorphisms, (for example knowledge of functional connections between the rodent bed nucleus of the stria terminalis and anteroventral periventricular nucleus, or the zebra finch high vocal center and robust nucleus of the archopallium), progress has been made in determining site of action.

Sex differences in the SNB result from androgenic action on many morphological aspects of motoneuron and muscle (reviewed thoroughly in [6]). Site of androgen action for at least some of these features has been determined, although apparently contradictory evidence exists (Table1). We will restrict our discussion of site of action to recent experiments that shed light on the cellular basis of androgenic organization of SNB motoneuron number and androgenic activation of SNB soma size and dendritic extent.

**Table 1:** Site of action in the spinal nucleus of the bulbocavernosus

**Evidence**	**Site of action**	**Reference**
**Organization of motoneuron number**		
Androgens can attenuate cell death in cultured motoneurons	Motoneuron	[27]
Androgen receptor is expressed in muscle but not motoneuron during critical period	Muscle, not motoneuron	[28][26]
Ablation of spinal cord containing SNB does not prevent masculinization of target muscle	Not motoneuron	[29]
Ablation of target muscle prevents masculinization of SNB motoneurons	Muscle	[30]
Anti-androgen delivered to target muscles prevent masculinization of SNB motoneurons	Muscle	[31]
Administering CNTFa, trkB or trkC antagonists to target muscle more effective than systemic administration in preventing masculinization of SNB motoneurons	Muscle	[32]
SNB motoneurons of androgenized X^*Tfm*^/X^*wt*^ females survive regardless of functional *Ar* within motoneurons	Not motoneuron	[33]
Selective genetic muscle *AR* replacement does not masculinize SNB of *Tfm* rats	Not only myocytes	[34]
Selective genetic deletion of Ar in myocytes reduces but does not prevent masculinization of SNB	Not only myocytes	[35,36]
**Organization of motoneuron size**		
Neonatal axotomy of SNB motoneurons prevents normal soma size	Muscle	[37]
**Organization of motoneuron dendritic extent**		
ER agonist/antagonist delivered to target muscles more effective that systemic delivery in regulating dentritic outgrowth of SNB motoneurons	Muscle	[38]
**Activation of motoneuron size**		
Axotomy of SNB motoneurons prevents androgenic maintenance of SNB motoneuron size	Muscle	[39-41]
AR agonists or antagonists delivered unilaterally to target muscles do not affect ipsilateral motoneurons	Motoneuron	[42]
Size of SNB motoneurons of androgenized X^*Tfm*^/X^*wt*^ females depends on functional *Ar* within motoneurons	Motoneuron	[43]
Selective overexpression of *AR* in myocytes does not result in increases in quadriceps motoneuron size	Not myocyte	[44]
**Activation of motoneuron gene expression**		
Local injection of target muscle extracts from castrated males increases CGRP in SNB motoneurons	Muscle	[45,46]
Axotomy of SNB motoneurons affects AR	Muscle	[47][39][48]
CGRP, CDH2 in SNB motoneurons of androgenized X*Tfm*/X*wt* females depends on functional *Ar* within motoneurons	Motoneuron	[49,50]
**Activation of dendritic extent**		
AR agonists or antagonists delivered unilaterally to target muscles do affect ipsilateral motoneurons	Muscle	[42]
Selective overexpression of *AR* in myocytes results in androgen-dependent increases in dendritic extent in quadriceps motoneurons	Myocyte	[44]

Androgenic action on the SNB system is almost exclusively mediated by ARs. This conclusion comes partly from the study of rats with the testicular feminization mutation (*Tfm* see [51] for review), which is a loss of function *Ar* mutation [52]. The *Ar* gene is located on the X chromosome, allowing for some interesting experimental preparations. Genetic *Tfm* male rats (X^*Tfm*^Y) have a complete loss of *Ar* function, but produce high levels of testosterone. These *Tfm* males have a feminine SNB system on all measures examined, including motoneuron number [15,34], size [15,34], and size of target muscles [34,53]. Female carriers of the *Tfm* allele (X^*Tfm*^X^wt^) have also been used to address site of action in the androgenic masculinization of the SNB system. Due to X chromosome inactivation, and indirect rescue of SNB motoneurons, androgenized females possess a mosaic of SNB motoneurons, approximately half of which express the X with the *Tfm* mutation while the remainder express the X chromosome with a wt *Ar* allele. Androgenic effects on SNB motoneurons that are direct should only occur in those cells expressing wt *Ar*, whereas indirect effects should occur in both cells types. This type of mosaic analysis has been used to provide evidence of direct activation of SNB soma size and gene expression via actions on motoneuronal *Ar*, and also that masculinization of SNB motoneurons occurs independently of motoneuronal *Ar* (Table1).

Genetic engineering makes more refined manipulations of *Ar* possible. For example, it is possible to manipulate a gene of interest in specific tissue types and/or developmental periods. We and our collaborators recently engaged in such studies to determine the role of *Ar* within myocytes in mediating various known actions of testosterone. Myocytes have widely been assumed to be the cell type on which androgens were acting to rescue the SNB neuromuscular system for a number of reasons: myocytes are the primary cell type in muscle; myocytes are the cells with which neurons functionally interact; retrograde neurotrophic influence of myocyte on motoneurons has been well characterized in other systems; and there is evidence of AR expression within myocytes [25,34].

We therefore attempted to rescue the *Tfm* SNB with selective myocyte *Ar* replacement*.* To do this, we used a human skeletal actin-AR *(HSA-AR*) transgene, which comprises a promoter from a skeletal myocyte-specific actin isoform (*ACTA1*) driving expression of AR, in rats [34]. The initial research question in this case was to determine whether AR expression restricted to myocytes would be sufficient to rescue the SNB of *Tfm* males. This experiment provided a strong test of the predictions made by the prevailing model of androgenic rescue of the SNB at that time, which held that testosterone acts on ARs within myocytes to masculinize the target muscles as well as rescuing SNB motoneurons via a retrograde neurotrophic mechanism. Much to our surprise, we were not able to verify either of these predictions. Instead, expressing AR in myocytes had no measurable masculinizing effect on either target muscles or motoneurons in rats [34]. This puzzling result might be explained either by myocyte AR not being involved in the organization of the SNB, or by AR in this cell type being but one of several necessary conditions. The former interpretation is somewhat supported by a mouse model in which *Ar* has been genetically deleted selectively within myocytes [35]. Despite the lack of myocyte *Ar*, these mice nonetheless possess a masculine (albeit hypomorphic) target musculature. Similarly, these mice have masculine, but reduced SNB motoneuron number [36]. One important caveat when interpreting the results of these experiments is that the efficiency of *Ar* knockout in this model is uncertain, both in terms of how completely *Ar* was deleted when measured in adulthood [35], and also whether *Ar* deletion occurred prior to the sensitive period for androgenic masculinization of the SNB. These results may therefore either be interpreted as being consistent with a necessary role of myocyte *Ar* in the androgenic masculinization of the SNB, or they may be interpreted as a modulatory role for myocyte *Ar* in this process.

On balance, these studies indicate that myocytes indeed play a role in the androgenic masculinization of the SNB, but they do not act alone. This conclusion raises the question of which cells are involved. Skeletal muscle is composed of a variety of cell types other than myocytes, including: adipocytes, endothelial cells, fibroblasts, satellite cells and Schwann cells. Perhaps because myocytes have been such an exclusive focus of research, we have limited knowledge of these other cell types as candidates. Among these cells, both Schwann cells [54,55] and satellite cells are androgen regulated [53,56-59]. There is some evidence for AR expression within satellite cells [60,61], but the best available evidence indicates that Schwann cells do not express AR *in situ* in nerve [62]. Unfortunately, we currently have no information concerning the possibility that AR is expressed by these cells within the target musculature in the critical period, nor have tests of AR function been made in these cells.

We and our collaborators also studied site of action in androgenic activation of SNB dendrites using *HSA-AR* rats. In this case, a rescue experiment concerning the SNB was not feasible, as *HSA-AR Tfm* males do not develop a masculine SNB [34]. Instead, we decided to explore an alternative possibility: that by making muscles artificially sensitive to androgens, we might consequently impart androgen sensitivity to spinal motoneurons that normally show little response to androgens. To test this idea, we studied motoneurons innervating quadriceps muscles. As expected, castration did not reduce dendritic extent of wt male quadriceps motoneurons [44]. In contrast, the dendritic extent of quadriceps motoneurons of transgenic *HSA-AR* males showed an enhanced response to androgen manipulations: sham-operated or castrated and androgen-maintained transgenic males had greater dendritic extent than sham-operated or androgen-maintained wt males. This effect was eliminated by castration without androgen maintenance, which equalized dendritic extent between wt and transgenic males [44]. Soma size was unaffected by androgen manipulations in either genotype [44]. These results bear out predictions made from studies performed in the normally androgen sensitive SNB: that myocytes are the site of action of testosterone in promoting dendritic extent but that testosterone acts on motoneurons themselves to promote increases in soma size.

These results most obviously support a neurotrophic mechanism whereby the peripheral cells (myocytes) increase dendritic extent of innervating motoneurons. Target musculature is suspected to exert retrograde neurotrophic influence via brain derived neurotrophic factor (BDNF) on androgenic activation of dendritic extent [63]. However, another mechanism whereby myocyte AR might affect motoneurons is suggested by our characterization of *HSA-AR* rats. We investigated the possibility that androgens can influence body composition via myocyte *AR*[64]. Androgens are known to increase lean body mass and decrease fat body mass. It was widely assumed that this regulation occurs in a cell autonomous manner within myocytes and adipocytes. Over-expression of *AR* within myocytes does indeed increase myocyte size, but curiously, it is also sufficient to decrease adipose tissue [64]. Because these alterations in body composition were associated with changes in resting metabolic rate, muscle respiration, and, in mice, alterations in mitochondrial morphology, these results are most obviously consistent with an endocrine mechanism of action [64,65]. It therefore remains possible, but it seems less likely, that endocrine mechanisms explain the effect of myocyte *AR* on spinal motoneurons.

### Extrinsic influences on SNB development

Although the main focus of study in the sexual differentiation of the SNB has been on an intrinsic factor (i.e. androgens), there is reason to believe that extrinsic factors may also contribute to this process. Interactions with conspecifics, and especially with the dam, have been investigated as potentially mediating some aspects of organization of the system. Maternal behavior directed towards pups is unequal between male and female pups [66]. The salient difference in maternal behavior is in anogenital licking, with dams licking male pups more than female pups. Eliminating this sex difference in licking, via maternal anosmia, interferes with the ability of adult males to accurately intromit [67,68]. Concomitant with this behavioral impairment, reduced masculinization of the SNB system is also observed, characterized by reduced motoneuron number [69], soma size, dendritic extent and target musculature [70]. It is unclear exactly how this extrinsic factor of maternal licking results in alterations in the SNB system, but the obvious mechanism would be via tactile sensory stimulation (i.e. a sensorineural mechanism).

This idea of a sensorineural mechanism mediating maternal effects on organization of the SNB is supported by several lines of evidence. Firstly, some of these effects of maternal anosmia can be reproduced by artificially rearing pups, and reversed by providing licking-like tactile stimulation [71]. Low levels of tactile stimulation in artificially reared pups results in a decrease in soma size, dendritic length of SNB motoneurons, lower target musculature weight and *ex copula* penile reflexes in adulthood, and higher levels of stimulation reversed these deficits. Secondly, perineal stimulation of neonatal males results in neural activity and oxytocin release in the vicinity of the SNB [72]. Nonetheless, a general endocrine mechanism cannot be discounted, as tactile stimulation of pups is associated with several endocrine events, including alteration in the regulation of glucocorticoids [73], central sensitivity to estrogens [74], and alterations in diverse hormonal systems regulating growth and energy balance [75]. It would be interesting to rule out the most obvious source of endocrine influence of tactile stimulation of pups by performing experiments in which licking (or tactile stimulation) was manipulated in pups in which testosterone levels were controlled by gonadectomy and dosed testosterone administration.

Extrinsic influences have also been implicated in activation of the SNB. Several studies have been conducted to evaluate the role of sexual stimulation on the SNB. In the first study, gonadally intact adult males were either given access to sexual partners, or only to sexual cues without coital contact, or given no social partners for 4 weeks [76]. In this case, no alteration in soma size or dendrites was observed. In another similar study, males were gonadectomized and maintained on an equal dose of testosterone and either given continual access to sexual partners or non-receptive females for 4 weeks, in which case decreased SNB soma size was observed [77]. However, despite the attempt to equalize testosterone between groups, this effect may nonetheless have been due to copulating males having less circulating testosterone [78]. It is unclear, then, whether the variations in sexual behavior one might reasonably expect in free-living rats would alter soma size or dendritic extent of SNB motoneurons, and if so, whether this would occur via a sensorineural or an endocrine mechanism.

### Applying insights from the SNB to other systems

One sometimes comes across a spoken or unspoken objection that the SNB is simply different from other sexual dimorphisms, and represents an exception to, rather than an example of, a CNS dimorphism. Certainly there are salient differences between the SNB and the few cases of brain dimorphisms about which we have some mechanistic understanding: the SNB is AR regulated rather than estrogen receptor regulated (although it is clear at this point that AR does indeed regulate other CNS dimorphisms [51]), the SNB is in the spinal cord rather than the brain, the SNB consists of primary motoneurons, rather than interneurons, and so on. These differences beg the question of whether we can generalize insights gained from the SNB to other neural dimorphisms. This last question has been affirmed convincingly by the SNB’s impressive track record of leading research into principles of neural differentiation [6]. In fact, the insights gained from studies of the organization of the SNB have already been used to propose indirect action on brain dimorphisms via the periphery [7].

### Interactions of other CNS dimorphisms with peripheral structures

#### *Tactile interactions*

In addition to its targeted effects on SNB morphology and sexual behaviour, maternal licking in general contributes to many other aspects of development, including reproductive behavior and stress response [66,73]. This line of research has produced some concrete demonstrations of extrinsic stimuli, presumably acting via tactile sensation, altering the development of the CNS [79]. For example, variations in licking by dams results in alterations in CNS regulation of the hypothalamic-pituitary-adrenal axis via alterations in hippocampal glucocorticoid receptor expression levels [73]. A more direct link to sexual differentiation is provided by the finding that variation in maternal licking contributes to pups’ future maternal behavior [73], which is highly sexually dimorphic. This peripheral contribution to sexual differentiation of behavior is plausibly linked to alterations in the CNS by the finding that maternal licking can alter estrogen receptor expression in the medial preoptic area when offspring of dams exhibiting extreme amounts of maternal licking are compared [74]. Although these alterations in neural steroid hormone receptor expression ultimately occur via epigenetic mechanisms [80], as with the SNB, it is unclear whether tactile influence on CNS is mediated via sensorineural mechanisms, or endocrine mechanisms, or both.

#### *Chemosensory interactions*

Historically, the argument has been made (e.g. [81]) that sexually differentiated behavior, such as copulation, might result primarily from sex differences in the organs necessary for those behaviors (the genitals in the case of copulation). In essence, this argument proposes that the brain is more or less a *tabula rasa* upon which sexual differentiation of behavior is a consequence of unequal sensation and experience produced by sex differences in peripheral structures. For example, according to this argument, males are better able to engage in male-typical insertive sexual behaviors because they possess intromissive genitals.

More recently, a similar argument was made on empirical grounds [82,83]. In this study, female mice whose vomeronasal organ (VNO), a peripheral neural structure, was surgically removed, or female mice with genetic ablation of *Trpc2*, an ion channel mediating VNO chemosensation, showed increased male typical copulatory behavior (mounting). The authors concluded that females therefore possess the neural circuits necessary for male typical copulation and that the VNO (and specifically the TRPC2 channel) serve to suppress these behavioral responses. The authors go on to speculate that sex differences in behavior may generally arise from sexually differentiated peripheral structures, with both sexes retaining a sexually bipotential brain throughout life [83]. In both of these cases, the assertion that sex differences in behavior result solely from sex differences in peripheral structures has been dismissed, primarily based on overwhelming evidence of sex differences in the structure of the CNS (for example, see [84]), and also extensive evidence that, especially in the case of copulation, sex-typicality of copulatory behavior can be dissociated from sex-typicality of genitals (e.g. [1,85]). Furthermore, there is reason to think that the VNO ablation used to study this question [82] may have had unintended consequences, as others have not observed similar behavioral effects of this surgery [84], and observed effects might affect behavior via alterations in testosterone [86].

Sex differences in behavior and/or neural structure have been seen as relative, rather than absolute, from the earliest study [1]. From this perspective, it is unsurprising to observe “male” copulatory behavior in females. Indeed, mounting is likely a normative element of the feminine behavioral repertoire, at least in rats [87]. However, this relative sex difference in copulatory behavior should not be taken as evidence that the brain is not differentiated.

Nonetheless, there is good reason to think that the VNO is important for sexual differention of brain and behavior. There is evidence that the for sexually dimorphic VNO [88] and main olfactory epithelium [84] processing of chemosensory cues. We might imagine therefore that these peripheral chemosensory structures, either via neurotrophic and/or sensorineural mechanisms might contribute to sexual differentiation of the extended olfactory system, which includes many of the classic sexual dimorphisms that are popular subjects of study, including the medial amygdala posterior division, bed nucleus of the stria terminalis and the anteroventral periventricular nucleus. It is interesting that dihydrotestosterone administration to neonatal females results in a masculine style of bedding preference [89]. Although there is no clear indication regarding the mechanism of this behavioral effect, dihydrotestosterone is generally ineffective in producing CNS dimorphisms. One potential explanation therefore is that dihydrotestosterone masculinizes bedding preference via actions on peripheral structures, such as the VNO and/or accessory olfactory epithelium. Although this hypothesis is certainly speculative at this point, it might help explain some other recent findings in *Ar* mutant mice (described below) that are puzzling according to a traditional view of exclusively local differentiation of the brain.

Gonadotropin releasing hormone neurons are another set of peripheral cells that interact with, and in fact contribute to, sexually dimorphic CNS structures. These neurons migrate into the CNS from the primordia of the VNO [90] beginning at 10.5 days post coitus in mice [91], prior to gonadal differentiation. We are unaware of any data that indicates sexual differentiation in this migratory process, or of influence of these neurons on other sexually dimorphic cell populations other than their obligatory role in gonadal hormone production. Nonetheless, adult gonadotropin releasing hormone neurons are sexually dimorphic [92], as is their regulation [93], and these migratory neurons remain a potential source of peripheral influence on the developing CNS.

#### *Effects of ar manipulation on brain dimorphisms and behavior*

*Tfm* mice [94] exhibit profound deficits in copulatory and sociosexual behaviors (see [51] for review). Similar to *Tfm* mutants, males with global genetic deletion of *Ar* exhibit profound deficits in masculine sexual behavior, even following treatment with estrogens or androgens [95]. However, as others have argued [51], we must be somewhat cautious when interpreting results from studies with *Tfm* or equivalent mutants, precisely because they lack the peripheral structures (masculine genitals) that are essential for some classic measures of behavioral masculinization (e.g. intromission and ejaculation, although the associated gross motor patterns may be observed infrequently in individuals with feminine genitals [85]), and also because neural ARs regulate brain aromatase, and loss of *Ar* function alters androgen production.

More striking is that selective genetic deletion of *Ar* in neurons seems to reproduce only a relatively mild version of the copulatory deficits observed in loss of function *Ar* mutations such as *Tfm* or global *Ar* knockout [96,97]. In these experiments, *Ar* was inactivated selectively in neurons using a *Nes* promoter. Both studies report a reduction in the proportion of mutant males mating with stimulus females and attacking intruder males relative to wt male controls. In one study an increase in latency to initiate intromissions, thrusts and ejaculations was observed even in males that did copulate [96]. In the other case, no group differences in copulatory behaviors were observed in mice which copulated are included [97]. It is unfortunate that data from females was not presented, making it difficult to assess the degree of sexual differentiation in mutant males. We should also bear in mind that this results may underestimate the improtance of neural *Ar* for sexual differentiation, because of potential problems with the efficiency of *Ar* deletion discussed previously.

On balance, these studies suggest that neural deletion of AR interferes with masculinization of copulatory behavior, although it does not prevent it entirely in the manner of global loss of function of *Ar*. This suggests that ARs other than those in neurons (and presumably those outside the CNS) mediate the essential features of sexual differentiation of mouse copulatory behavior. If we extend this thinking, we might also suspect that other sociosexual deficits exhibited by *Tfm* or *Ar* knockout animals might result from other peripheral deficits in masculinization of, for example, genitals, chemosensation, or pheromone production.

## Conclusions

The principle of accepting the simplest explanation for phenomena is undeniably useful in science. In the case of neural dimorphisms that are caused by androgens, the simplest explanation is of course that androgens act directly on the cell populations in question. Perhaps unfortunately for those who study this problem, when this assumption was put to the test for the first time, using the SNB, it proved inconsistent with the data. The results of studies in the SNB have identified at least three potential mechanisms whereby the periphery might influence the CNS: endocrine, neurotrophic and sensorineural. The less intuitive explanation, that differentiation occurs indirectly, and in some cases via peripheral structures, therefore becomes a necessary complexity in at least some cases.

However, it is not worth abandoning the hypothesis that androgens contribute to CNS sex differences directly via actions on neurons. Other studies of the SNB, including those dealing with site of androgen action in activating soma size and gene expression, have yielded results that are consistent with this hypothesis. Furthermore, the site of action in androgenic differentiation of any brain region is simply unknown and so the question remains open. Finally, there are findings that suggest local, neural effects, such as the lack of masculinization of aggressive behavior in males with selective genetic deletion of *Ar* within neurons [97].

In the end, the answer is unlikely to be that *either* the periphery *or* central mechanisms explain sexual differentiation of the CNS. Rather, as has been the case for the SNB, both peripheral and central mechanisms contribute to any given sex difference. Multiple mechanisms can come about because there are multiple sexually differentiated features, each of which may have a different mechanism of action. For example, SNB motoneuron number is determined by androgen action on muscle whereas some gene expression appears to be determined by androgen action directly on SNB motoneurons (see Table1). Alternatively, an individual sexually differentiated feature may have both peripheral and central contributions. For example, there is evidence for androgen action on both muscle and SNB motoneurons to determine SNB soma size.

Fortunately, there is promise for distinguishing between these various possibilities. Methodology in which androgenic effects on central and peripheral structures are dissociated using genetic engineering are becoming increasingly available (e.g. [98]), and we anticipate that genetic approaches to the site of action question will provide further examples of the complex relationship between the CNS and the periphery during sexual differentiation.

## Abbreviations

AR, androgen receptor; BDNF, brain derived neurotrophic factor; CNS, central nervous system; SNB, spinal nucleus of the bulbocavernosus; Tfm, testicular feminization mutation; VNO, vomeronasal organ; wt, wild type.

## Competing interests

The authors have no competing interests to declare.

## Authors’ contributions

All authors were involved in the conception of the review, as well as drafting and revising it. All authors read and approved the manuscript.
